# Smile Aesthetics Assessment in Patients Undergoing the Finishing Phase of Orthodontic Treatment: An Observational Cross-Sectional Study

**DOI:** 10.7759/cureus.45644

**Published:** 2023-09-20

**Authors:** Daniel Arrubla-Escobar, Diana M Barbosa-Liz, Oscar Zapata-Noreña, Alvaro Carvajal-Flórez, Karina Correa-Mullet, Sandra L Gómez-Gómez, Carlos M Ardila

**Affiliations:** 1 Basic Sciences, Institución Universitaria Visión de Las Americas, Medellin, COL; 2 Basic Sciences, GIONORTO Research Group, Faculty of Dentistry, Universidad de Antioquia, Medellin, COL; 3 School of Dentistry, Universidad de Antioquia, Medellin, COL; 4 Basic Sciences, Faculty of Dentistry, Universidad de Antioquia, Medellin, COL; 5 Basic Sciences, Biomedical Stomatology Research Group, Universidad de Antioquia, Medellin, COL

**Keywords:** malocclusion, dental esthetics, treatment, orthodontics, smiling

## Abstract

Objective

To describe the smile characteristics of patients entering the finishing phase of orthodontic treatment.

Methods

This observational study involved a non-probabilistic sample of 48 patients. Clinical records served as the basis for determining the type of treatment (with or without extractions). Photographs were analyzed to obtain smile variables. Dental casts and panoramic radiographs were evaluated to ascertain the cast-radiograph evaluation (CRE) index. Univariate and bivariate analyses were conducted at a significance level of 0.05.

Results

The study evaluated 24 men and 24 women, with an average age of 20.10 ± 6.78 years. Fifty percent of the patients did not undergo extractions, and the average CRE index for the sample was 34.83 ± 9.01. Regarding the smile, a medium smile line was prevalent in 66.7% of cases, and a non-consonant smile arc was observed in 58.3%. Significant differences in the smile arc were found between patients with and without extractions (p=0.019). Right and left buccal corridors measured 2.52 mm ± 1.52 and 2.43 mm ± 1.37, respectively. The upper dental midline deviated by 0.80 ± 0.91 mm and had an angulation of 1.65 ± 2.05º. Both variables showed significant differences between Class I and Class II patients (p=0.020; p=0.027). Symmetrical smiles were also observed (1.05 ± 0.17).

Conclusions

Based on our findings, clinicians should focus on the smile arc in patients who have not undergone extractions and on the midline inclination in Class II patients. These appear to be the most common areas for improvement in patients who are in the finishing phase of treatment. Additionally, considerable variability exists in the smile characteristics of patients still undergoing orthodontic treatment, leaving room for further enhancement of results.

## Introduction

Today, the smile is considered one of the most important aspects of facial appeal [1-3]; therefore, orthodontic treatment should ensure a harmonious smile, among other objectives [4,5]. The parameters of a pleasant or aesthetic smile were first developed by Hulsey [6] and then clearly defined and determined [7,8]. Moreover, some authors have shown that there is a relationship between dental position and the appearance of the soft tissues in a smile [9,10].

Likewise, efforts have been made over the years to obtain optimal aesthetic and occlusal results in orthodontic treatments. Among these efforts is the use of indexes that allow for quantifying the achievement of treatment objectives, i.e., the grading system for scoring dental casts and panoramic radiographs (CRE) developed by the American Board of Orthodontics (ABO) [11].

However, there are well-treated cases where the occlusal evaluation shows that they meet all the ABO criteria but do not necessarily reproduce an aesthetic smile [1,7]. In this sense, it is imperative that orthodontists recognize the positive elements that make up a harmonic smile (e.g., smile line, smile arc, buccal corridors, midline deviation, angulation of the midline, and symmetry of the smile) and create strategies to optimize the attributes that are objectively outside the desired aesthetic parameters, dedicating themselves to achieving them with greater care in the finishing phase [12,13], where there is still an opportunity to improve.

In orthodontic diagnosis, the aesthetic evaluation is made in a posed smile, and the relationships between the teeth, the labial frame, and the gingival architecture are evaluated [14]. Some criteria have had greater relevance and impact on smile aesthetics [13,15] and can be evaluated objectively. With the purpose of protecting the smile arch and achieving more aesthetic results from the beginning of the treatment, Pitts in 2017 suggested positioning the lateral and central braces more gingivally (Smile Arch Protection) [16]. However, at the end of the treatment phase (usually six months before appliance removal), it is necessary to evaluate whether the characteristics of the smile meet the proposed objectives or if any mechanism should be implemented to achieve them.

Considering that during the finishing phase it is necessary to optimize the aesthetic, functional, and occlusal results and seeing that clinical studies have been mainly carried out in finished patients, the objective of this study was to describe the smile characteristics of the patients who started the finishing phase of orthodontic treatment in an orthodontics graduate program from a Latin American dental school.

## Materials and methods

An observational cross-sectional retrospective study was conducted, and the non-probabilistic sample consisted of 48 subjects who met the inclusion criteria: being in the finishing phase of orthodontic treatment between 2013 and 2018 at the Orthodontics Postgraduate Program at the University of Antioquia, UdeA (Medellín, Colombia), and having standardized records, used by the UdeA Finishing Protocol [17].

It is important to emphasize that the sample size was determined by convenience, and we selected all patients who met the selection criteria during the established period. However, it is worth noting that power estimation at the conclusion of the study, with the set number of cases (n=48) and the specified outcomes (CRE), did indeed achieve a power of 80%. In this context, considering an average difference of 2.75 in the CRE variable between participants with and without extractions, and an observed standard deviation of 2, plus a power of 80% and a significance level of 0.01, the number of participants should be 16 individuals per group. Thus, our sample size greatly exceeds the number of participants obtained in the sample calculation.

Patients requiring prosthetic-periodontal and/or surgical treatments, or who presented any craniofacial syndrome or anomalies, were excluded. The present study received approval from the Ethics Committee of the Faculty of Dentistry at the University of Antioquia (08-207, #2-2017).

Standardized records were obtained at a recognized radiological center (Maxillofacial Images S.A. Medellin, Colombia). Sex, age, type of treatment, and ANB angle were registered [13]. Posed smile photographs were analyzed using the Adobe Photoshop CS6® system, standardized at a 1:1 ratio. The variables evaluated in the photographs were: smile arc, smile line, buccal corridors, distance from the upper dental midline to the facial midline, midline angulation, and smile symmetry.

**Figure 1:**
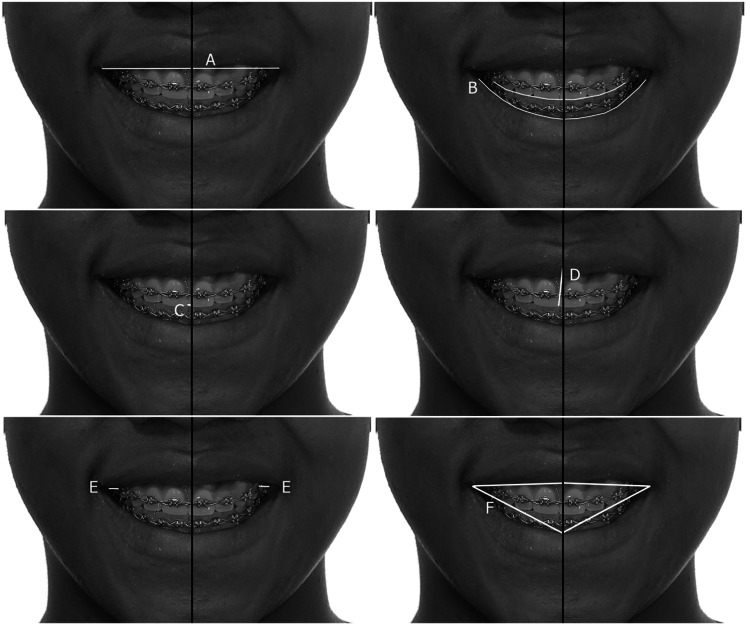
Guidelines for the measurement of variables (A) Smile line; (B) smile arc; (C) distance dental midline to facial midline; (D) angulation of dental midline to facial midline; (E) buccal corridors; (F) smile symmetry

The smile line was classified as high when the entire clinical crown of the anterior teeth was exposed; medium when 75-100% of the crown of the tooth was exposed, and low if less than 75% of the dental crown was exposed [18,19]. The smile arc was recorded as consonant or non-consonant [7]. The buccal corridors [1], the distance from the maxillary midline to the facial line, the angulation of the midline, and the symmetry of the smile [6] are described in Table 1 and Figure 1.

Records were evaluated to obtain the Cast-Radiograph Evaluation (CRE) index in accordance with the guide created by the ABO and adapted to the protocols of the University of Antioquia [11,20]. The eight criteria outlined by the CRE-ABO were measured using the instrument designed for this purpose. These criteria were alignment, marginal ridges, occlusal relationships, bucco-lingual inclination, interproximal contacts, overjet, root parallelism, and occlusal contacts. Following ABO guidelines, this variable was categorized based on the total score obtained as Excellent (≤20 points), Acceptable (21-27 points), or Less Than Acceptable (>27 points) [11].

To mitigate intra- and inter-operator variability, two experienced orthodontists (D.A. and O.Z.) repeated the measurements until both the kappa coefficient and intraclass correlation coefficient exceeded 0.8 for qualitative and quantitative variables, respectively. These data indicate an excellent level of agreement between the examiners. The information was analyzed using the IBM SPSS Statistics for Windows, Version 21 (Released 2012; IBM Corp., Armonk, New York, United States).

Statistical analysis

The Kolmogorov-Smirnov test obtained a normal distribution. For the bivariate analysis, the Pearson correlation analysis and the t-test for independent variables were used. ANOVA and Bonferroni post-hoc tests were used to determine the association of numerical variables with variables in more than two groups, and the chi-square test was used to explore the association between qualitative variables (significance level < 0.05).

**Table 1:** Definition of smile variables evaluated

Variable	Definition	Description
Smile arc	Relationship of the curvature of the incisal edges of the upper incisors and the canines with the curvature of the lower lip.	Consonant/non-consonant
Smile line	Amount of exposure from the lower edge of the upper lip to the incisal edge of the upper anterior teeth, drawing a horizontal line of reference for the lower margin of the upper lip and another by the incisal edge of the upper and/or lateral central incisors.	High/medium/low
Buccal corridors	The horizontal distance between the vestibular surface of the last visible maxillary tooth and the inside of the cheeks.	In millimeters
Distance from the maxillary midline to the facial line	The horizontal distance from the maxillary dental midline to the facial midline is determined by the lowest edge of the cupid arch and the center of the philtrum of the upper lip.	In millimeters
Angulation of the midline	The angle is formed by the dental midline and a vertical line parallel to the facial midline.	In degrees
The symmetry of the smile	The proportion between the areas formed from the commissures to the midpoints of the upper and lower lips. These points were considered: RC: right commissure; LC: left commissure; ULC: upper lip center; LLC: lower lip center. The symmetry is given by the formula. Symmetry = (RC - ULC) × (RC - LLC)LC - ULC × (LC - LLC).	Values greater than 1 indicate the predominance of the right side; values lower than 1 indicate the predominance of the left side

**Figure 2:**
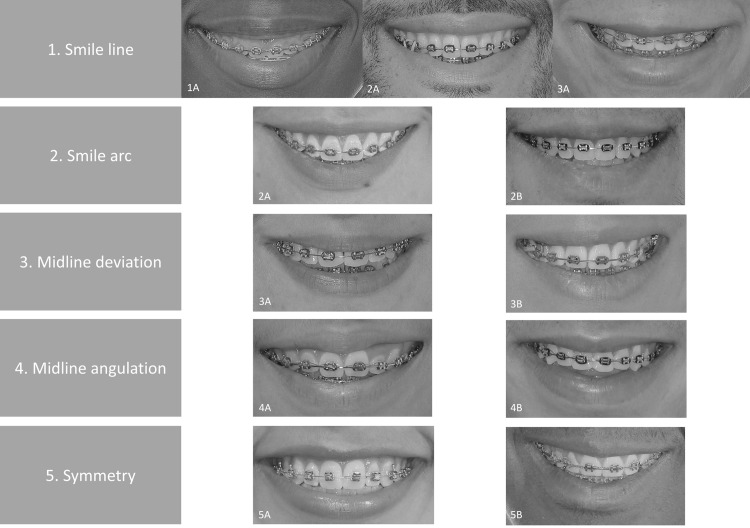
Evaluated smile variables representation 1. Smile line: 1A. High, 1B. Medium, 1C. Low. 2. Smile arc: 2A. Consonant, 2B. Non-consonant. 3. Midline deviation: 3A. Centered upper dental midline, 3B. Upper dental midline deviated 3.8 mm to the right. 4. Midline angulation: 4A. Midline with no angulation, 4B. Upper dental midline angulated 5° to the right. 5. Symmetry: 5A. Symmetry value = 1.0 (symmetrical), 5B. Symmetry value = 1.2 (smile with predominance of the right side)

Figure 2 provides examples of the variables and categories mentioned.

## Results

The sample included 48 patients (50% men and 50% women; 20.10 ± 6.78 years). Fifty percent of the sample (n=24) were without extractions. Extraction treatments were more prevalent in women (n=15, 62.5%) than in men (n=9, 37.5%). Class I and II patients constituted 91.7% (n=44) of the total subjects. At the start of the finishing phase, 79.2% (n=38) of patients obtained a CRE score higher than 27 points (Table 2).

**Table 2:** Demographic characteristics of the sample (n = 48). The absolute values (n) and their respective percentages (%) are presented, accompanied by the mean value and the standard deviation (SD). CRE: cast-radiograph evaluation index.

Variable	Value
Sex, n (%)	
Male	24 (50.0)
Female	24 (50.0)
Age in years, mean ± SD	20.10 ± 6.78
Age group, n (%)	
<16 years	7 (14.6)
16–18 years	19 (39.6)
19–22 years	13 (27.1)
>22 years	9 (18.8)
Number of extractions, mean ± SD	1.92 ± 1.98
Extractions, n (%)	
Yes	24 (50.0)
No	24 (50.0)
ANB, mean ± SD	3.81 ± 2.46
Sagittal relationships, n (%)	
Class I	23 (47.9)
Class II	21 (43.8)
Class III	4 (8.3)
CRE score, mean ± SD	34.83 ± 9.01
CRE, n (%)	
Less than acceptable	38 (79.2)
Acceptable	9 (18.8)
Excellent	1 (2.1)

A medium smile line was the most frequently encountered among the study participants, regardless of gender, age, sagittal relationships, or treatment performed (with or without extractions). Likewise, the smile arc was non-consonant in 58.3% (n=28) of the sample. There was a statistically significant difference (p=0.019) between the extraction and non-extraction groups, showing non-consonant smile arcs in the non-extraction group (75%; n=18). Figure 2 shows a female patient who underwent premolar extractions for treatment and exhibited a consonant smile (2A), and a male patient who underwent no extractions and exhibited a non-consonant smile (2B). In this study, in relation to the smile line and smile arc, no statistically significant differences were found for gender, age, and malocclusion (Table 3).

**Table 3:** Comparison of the smile line and smile arc with demographic variables studied (n = 48). The absolute values (n) and their respective percentages (%) are presented. Values p<0.05 were considered statistically significant. CRE: cast-radiograph evaluation index.

Variable	Smile line			p		Smile arc			p		
	High	Medium	Low				Non-consonant	Consonant			
Total, n (%)	7 (14.6)	32 (66.7)	9 (18.8)				28 (58.3)	20 (41.7)			
Sex, n (%)											
Male	5 (20.8)	15 (62.5)	4 (16.7)		0.467		15 (62.5)	9 (37.5)		0.558	
Female	2 (8.3)	17 (70.8)	5 (20.8)				13 (54.2)	11 (45.8)			
Age group, n (%)											
<16 years	3 (42.9)	4 (57.1)	0 (0.0)		0.299		4 (57.1)	3 (42.9)		0.903	
16–18 years	2 (10.5)	13 (68.4)	4 (21.1)				10 (52.6)	9 (47.4)			
19–22 years	2 (15.4)	8 (61.5)	3 (23.1)				8 (61.5)	5 (38.5)			
>22 years	0 (0.0)	7 (77.8)	2 (22.2)				6 (66.7)	3 (33.3)			
Sagittal relationships, n (%)											
Class I	3 (13)	14 (60.9)	6 (26.1)		0.699		14 (60.1)	9 (39.1)		0.662	
Class II	3 (14.3)	15 (71.4)	3 (14.3)				11 (52.4)	10 (47.6)			
Class III	1 (25.0)	3 (75.0)	0 (0.0)				3 (75.0)	1 (25.0)			
Extractions, n (%)											
Yes	2 (8.3)	16 (66.7)	6 (25)		0.319		10 (41.7)	14 (58.3)		0.019	
No	3 (12.5)	16 (66.7)	5 (20.8)				18 (75)	6 (25)			
CRE, n (%)											
Less than acceptable	6 (15.8)	26 (68.4)	6 (15.8)		0.737		23 (60.5)	15 (39.5)		0,471	
Acceptable	1 (11.1)	5 (55.6)	3 (33.3)				4 (44.4)	5 (55.6)			
Excellent	0 (0.0)	1 (100)	0 (0.0)				1 (100)	0 (0.0)			

The mean of the right and left buccal corridors were 2.52 ± 1.52 mm and 2.43 ± 1.37 mm, respectively. A maxillary dental midline deviation of 0.80 ± 0.9 was found. Statistically significant differences were found between Class I and II, evidencing a greater deviation of 0.92 mm in Class II patients (p =0.002). Likewise, a statistically significant (p =0.027) angulation of the maxillary dental midline concerning the facial line of 1.65 ± 2.05° was found. This angulation was 1.63° higher for Class II than Class I. Finally, the results showed the presence of symmetrical smiles with a proportion value of 1.05 ± 0.17. Table 4 describes these findings in detail.

**Table 4:** Quantitative smile characteristics of the sample (n = 48). The absolute values (n) and their respective percentages (%) are presented, accompanied by the mean value and the standard deviation (SD). Values p<0.05 were considered statistically significant. RBC: right buccal corridor; LBC: left buccal corridor; MDMF: maxillary dental middle line distance to facial midline; MDMA: maxillary dental midline angulation; S: symmetry of the smile; CRE: cast-radiograph evaluation index.

Variable	RBC mm	p	LBC mm	p	MDMF mm	p	MDMA degrees	p	S	p
Total, mean ± SD	2.52 ± 1.52		2.43 ± 1.37		0.80 ± 0.91		1.65 ± 2.05		1.05 ± 0.17	
Sex, mean ± SD										
Male	2.74 ± 1.51	0.3	2.68 ± 1.45	0.2	0.86 ± 0.80	0.6	1.40 ± 1.30	0.4	1.04 ± 0.19	0.7
Female	2.30 ± 1.53		2.18 ± 1.27		0.74 ± 1.02		1.90 ± 2.60		1.06 ± 0.15	
Age group, mean ± SD										
<16 years	2.77 ± 1.94	0.8	2.64 ± 1.62	0.8	0.79 ± 1.07	0.3	1.04 ± 1.05	0.2	1.07 ± 0.18	0.1
16–18 years	2.58 ± 1.39		2.25 ± 1.50		0.85 ± 0.97		1.55 ± 1.56		1.01 ± 1.50	
19–22 years	2.55 ± 1.81		2.37 ± 1.43		0.46 ± 0.69		1.33 ± 1.82		1.14 ± 0.18	
>22 years	2.14 ± 1.11		2.76 ± 0.85		1.20 ± 0.89		2.81 ± 3.37		1.00 ± 1.63	
Extractions, mean ± SD										
Yes	2.43 ± 1.41	0.6	2.54 ± 1.34	0.6	0.83 ± 0.92	0.8	1.76 ± 1.60	0.7	1.06 ± 0.15	0.9
No	2.61 ± 1.64		2.33 ± 1.43		0.78 ± 0.92		1.55 ± 2.45		1.05 ± 0.19	
Sagittal relationships, mean ± SD										
Class I	2.44 ± 1.86	0.9	2.41 ± 1.66	0.9	0.39 ± 0.56	0.002	0.88 ± 1.54	0.02	1.08 ± 0.19	0.2
Class II	2.63 ± 1.25		2.43 ± 1.15		1.31 ± 1.02		2.51 ± 2.31		1.05 ± 1.14	
Class III	2.40 ± 0.45		2.60 ± 0.64		0.53 ± 0.64		1.55 ± 1.80		0.91 ± 0.08	
CRE, mean ± SD										
Less than acceptable	2.66 ± 1.57	0.6	2.49 ± 1.28	0.9	0.78 ± 0.97	0.9	1.59 ± 2.18	0.9	1.07 ± 0.17	0.6
Acceptable	2.08 ± 1.31		2.29 ± 1.82		0.77 ± 0.55		1.96 ± 1.62		1.01 ± 0.17	
Excellent	1.30 ± 0.00		1.70 ± 0.00		2.00 ± 0.00		1.40 ± 0.00		0.95 ± 0.00	

## Discussion

During the finishing phase of orthodontic treatment, orthodontists aim to optimize the aesthetic, functional, and occlusal results before the removal of appliances [21-23]. This study aimed to describe the characteristics of the smile evaluated in patients who had entered this phase of treatment. Considering that at this stage it is still possible to implement mechanics to make corrections that promote optimal treatment results, clinicians must know the aesthetic aspects that should be improved in the smile [22,24] before appliance removal. Professionals should identify the positive elements of each patient and establish strategies that can be used to maintain them, as well as determine those that are outside the desired parameters and should be improved [5,15]. The objective of this study was to identify the most common aspects of the smile that require improvement in patients who are in the finishing phase of orthodontic treatment, and to suggest therapeutic strategies for correcting these aspects. This information can assist clinicians in making the best therapeutic decisions for each case. In the patients studied, smile parameters were evaluated and aspects requiring improvement were corrected by the clinician within 6 months, as documented in the literature [25].

The present study analyzed variables that have been reported in previous studies as influencing the perception of the smile, such as the smile line, smile arc, buccal corridors, deviation from the upper dental midline, upper midline inclination, and symmetry of the smile [26-28]. The smile line is considered optimal when the upper lip reaches the gingival margin, showing the total cervical-incisal length of the central incisor, along with the interproximal gingiva [8]. In this study, this type of smile was classified as medium [19], indicating that at this point in treatment, the clinician had already reached this goal for many patients. According to McLeod et al., laypeople find a gingival exposure of between -2.5 and 4 mm to be acceptable [29]. Some studies have indicated that the smile arc is better perceived when it is consonant with the curvature of the lower lip [30]. Our study found a higher prevalence of non-consonant smile arcs (58.3%), showing a tendency to have a flat smile curve that can compromise the aesthetics of the smile [31,32]. This has also been reported by other researchers who have found that orthodontic treatment tends to conclude with flat smiles [6,7,33-35], sometimes due to standardized formulas for the positioning of braces [7,35]. Similarly, it has been described that the smile arc tends to be flatter in men than in women [36], and becomes flatter as age advances [8]. The findings of the present study confirm this information. The clinical implication of this finding suggests that clinicians should pay close attention to preserving the smile arc during braces positioning to prevent its flattening [16]. If the flattening of the smile arc has occurred, the braces should be reset to achieve an improvement in this variable.

On the other hand, the accepted amplitude for the buccal corridors ranges from 8% to 22% of the total smile width, or from 5.0 to 16 mm [30,37], with an ideal value being 11.6 mm [30]. Other studies performed on patients who had received orthodontic treatments showed buccal corridors of 5.0 ± 1.6 mm and 4.5 ± 1.3 mm [1]. In this study, averages of 2.52 ± 1.52 mm and 2.43 ± 1.37 mm were found for the right and left buccal corridors, respectively. A previous study showed a preference for broad smiles with narrow to medium buccal corridors, which ranged from 0% to 10% of the total smile width [38]. The corridors found in this study were smaller. However, it should be noted that different studies have found that buccal corridors do not significantly affect smile aesthetics [6,8,37,39].

The upper dental midline is an important focal point in an aesthetic smile [8]. A deviation of 0 mm is considered the ideal value; however, some reports show that a variation of up to 2.9 mm can be tolerable for laypeople [30]. In a systematic review [40], it was concluded that 2.38 mm is a tolerable deviation for this criterion. Herein, the deviation of the upper dental midline was 0.80 ± 0.91 mm, which falls in the tolerable range. Other studies found no difference in the perception of an ideal smile with deviations of 3 mm [37]. However, it is striking that there was a greater deviation of the midline for Class II patients. This aspect should be carefully evaluated, as well as the mechanism of midline correction including interproximal stripping, elastics, and others at this treatment phase [41].

Additionally, the parallelism in the inclination of the maxillary midline concerning the facial midline may be even more important than the coincidence between them [8]. The study by Ker et al. [30] showed that the deviation from the midline can be acceptable if the area of dental contact is vertical [8]. Moreover, Thomas et al. [42] proposed that an angle of 10° ± 6° is still tolerable. The present study obtained a dental midline angulation of 1.65° ± 2.05°, which is tolerable. However, the ideal would be to continue working on strategies for the correct inclination of the midline before finishing orthodontic treatment. This study also showed differences for Class II patients, which suggests that these patients seem to have more aesthetic needs than Class I-III patients. The position of the perioral soft tissues has a great influence on the symmetry of the smile [15] because it can directly affect the aesthetic results. This study found that there is a tendency towards the achievement of a value of 1.05 ± 0.17 for the proportion studied, like that reported by Hulsey [6], who stated that a smile should have a value close to 1.00 to be considered harmonic. This suggests that many of the aspects of smile asymmetry were corrected during the previous phases of orthodontic treatment.

CRE score was also considered in this study. Regarding occlusal characteristics, recent studies have shown that, at the end of treatment, the average scores of the CRE are close to 27.9 [43] and 31.41 [17]. This study obtained an average CRE score of 34.83 ± 9.01, giving 79.2% of the sample an unsatisfactory score for the ABO and CRE. However, it is important to bear in mind that these patients were beginning the finishing phase of their treatment, unlike those in studies of completed cases [44-46]. According to this, it is important to consider that occlusal aspects need to be evaluated and improved, if necessary, in the finishing phase. It also proposes that, if possible, a finishing protocol with strict follow-up should be implemented to achieve this objective [17,25,46-49]. Aesthetic results are directly related to patient satisfaction at the end of their treatment, and that may be a factor that negatively affects it [50].

It is important to note that several factors must be considered, including the implementation of strategies that allow clinicians to improve results and reduce the number of procedures required during the finishing phase, and the control of different factors from the previous phases, that can significantly diminish the duration of treatment and increase patient satisfaction [51,52]. Thus, considering the strategies that can contribute to a pleasant smile, guarantees the achievement of treatment objectives more efficiently. Based on the findings observed here, several of the evaluated characteristics were found to be within the parameters reported by the literature; however, there are still elements that can be perfected during finishing to achieve the desired aesthetic results. Table 5 presents some strategies that can be considered to improve the aesthetics of the patient's smile.

**Table 5:** Strategies for improving the aesthetics of the smile.

Characteristic	At the beginning of the treatment	During the treatment	Post-treatment
Smile arc	Proper positioning of braces.	Torque control of anterior teeth. Dental extrusion/intrusion. Repositioning braces. Orthognathic surgery.	Aesthetic management and rehabilitation of the anterior teeth.
Matched maxillary dental midline with facial	Determine the origin of the asymmetry. Proper positioning of braces Functional correction of the discrepancy: removal of premature contacts, maxillary expansion, functional appliances.	Deviation distalization, stripping, or asymmetric extractions according to the needs of the space. Asymmetric mechanics and constant control during the closure of spaces. Angulation Evaluate the inclination of incisors and the presence of the canted occlusal plane. Repositioning braces. Cantilevers for incisors uprighting. Surgical correction.	
Smile line	Proper positioning of braces.	Dental extrusion/intrusion. Orthognathic surgery.	Periodontal surgery. Application of botulinum toxin Infiltration of hyaluronic acid. Lip repositioning surgeries.
Buccal corridors	Rapid palatal expansion.	Transverse development with over-expanded arches. Belay-Titanium wire overlay Torque control of posterior segments with the help of intermaxillary elastics. Surgically assisted expansion.	
Smile symmetry	Myofunctional therapy. Esthetic management of perioral soft tissues.		

The mean age of the study sample was 20.10 ± 6-7 years. The most common smile line was a medium smile line, with 75-100% of the clinical crown exposed. This finding is consistent with what is expected in a population of this age. Several studies have shown that the smile line decreases with age due to soft tissue changes [53].

Although some authors have reported no differences in smile esthetics between the extraction and non-extraction groups, this study found significant differences when the smile arc was evaluated. In this case, the non-extraction group showed a higher prevalence of non-consonant arcs. This finding is consistent with the findings of Cheng et al. [1], who found that non-extraction treatment tends to increase maxillary incisor torque, flatten the smile arc, and reduce the incisor display. Recently, Ali et al. also reported differences in buccal corridor width and smile width ratio in patients with and without extractions [54].

Although the present study found no differences in relation to smile and the different malocclusions, other studies suggest a relationship between malocclusion and smile characteristics [55,56]. Kabalan et al. [56] found that skeletal Class II malocclusion influences the characteristics of the smile. Specifically, they found statistically significant differences in resting commissure height, resting upper lip thickness, incisor inclination, and smile arc between patients with Class I and Class II division 1 and 2 malocclusion. A possible explanation for the lack of a relationship between malocclusion and smile characteristics in the present study is the small sample size. This is a limitation of our study, as it could have led to a lack of power to detect a significant relationship.

Some limitations of this study must be recognized. Although the population of the study started with 209 patients, many of these did not meet the selection criterion, and some of them were discarded because the diagnostic records were not in good condition. For this reason, the study had a convenience sample of 48 patients. The small sample size limits the generalizability of the results. Future research should increase the population size. However, this study provides important insights into the aspects of smile improvement in patients who are in the final phase of orthodontic treatment. It is therefore important to consider that adequate storage management of diagnostic records is required, both for research purposes and for legal matters. The parameters used for the evaluation of the smile are multiple. In our study, we include those that we consider most relevant in the scientific literature. Moreover, this study did not assess muscle tonicity, a factor that influences several of the variables analyzed. Therefore, including a broader demographic range in the sample could provide a more comprehensive understanding of smile characteristics. This may entail considering aspects including age, ethnicity, and orthodontic treatment history. A longitudinal study tracking changes in smile characteristics over time could provide useful insights into the evolution of smiles over the orthodontic treatment process. Incorporating a patient satisfaction survey into the study could offer a subjective dimension. Understanding how patients see and feel about their smiles after therapy might provide useful information. Multivariate analysis approaches could be used to further investigate the correlations between various smile traits. This would allow for a more in-depth analysis of the elements that influence smiling outcomes. While the study reveals areas for improvement, orthodontists might benefit from therapeutic interventions or orthodontic procedures to address these concerns.

## Conclusions

The smile arc was significantly flattened in non-extraction patients. This finding suggests that strategies are needed to improve the smile arc in these patients or to avoid flattening it during orthodontic treatment. In addition, the variability in smile characteristics in patients still undergoing orthodontic treatment provides opportunities for improvement, and the orthodontist can tailor the therapeutic strategies to the specific needs of each patient, as proposed in the present study.
